# Commentary: Metabolites released from apoptotic cells act as tissue messengers

**DOI:** 10.3389/fimmu.2020.01878

**Published:** 2020-08-20

**Authors:** Chong Zeng, Zhongbao Shao, Jiwei Li, Hao Pan, Feiyue Xing

**Affiliations:** ^1^Medical Research Center, Shunde Hospital, Southern Medical University (The First People's Hospital of Shunde), Foshan, China; ^2^Department of Electronic Information Engineering, Guangzhou College of Technology and Business, Guangzhou, China; ^3^Department of Oncology, The First Affiliated Hospital of Zhengzhou University, Zhengzhou, China; ^4^Research Center for Integrative Medicine, School of Basic Medical Sciences, Guangzhou University of Chinese Medicine, Guangzhou, China; ^5^Institute of Tissue Transplantation and Immunology, Department of Immunobiology, Jinan University, Guangzhou, China; ^6^MOE Key Laboratory of Tumor Molecular Biology, Key Laboratory of Functional Protein Research of Guangdong, Higher Education Institutes, Jinan University, Guangzhou, China

**Keywords:** metabolites, apoptosis, pannexin 1, neighboring cell, gene expression

Apoptosis is a canonical form of regulated cell death that involves a series of biochemical events. Intriguingly, recent results revealed that apoptotic cells release metabolites that affect gene expression in healthy adjacent cells. Moreover, metabolite mixtures can ameliorate the specific inflammatory environment.

Cell death is a fundamental biological process by which multicellular organisms maintain cellular homeostasis. It is divided into two distinct processes: programmed cell death and uncontrolled cell death. Programmed cell death was first identified during investigations of insect development ~55 years ago (1). Our increased understanding of programmed cell death has led to great progress in the field of insect development. There are four types of programmed cell death: apoptosis, autophagy, pyroptosis, and ferroptosis (2). The first type of programmed cell death (apoptosis) is mediated by both caspase-dependent and caspase-independent pathways. Caspase-dependent apoptosis has been reported to regulate inflammation, cell proliferation, and tissue regeneration. However, with the rapid discovery of new mechanisms of apoptosis, the roles played by intracellular changes in apoptotic cells and their metabolites are not entirely clear.

Studies of caspase-dependent apoptosis revealed the existence of two major types of pathways for apoptosis: the intrinsic and extrinsic pathways (1). The extrinsic pathway can be triggered when death-related ligands such as tumor necrosis factor-α (TNF-α) or Fas ligand (FASL) bind to death receptors on the surface of their target cells. Subsequently, certain adaptor proteins [Fas-associated protein with death domain (FADD) and tumor necrosis factor receptor 1-associated death domain protein (TRADD)] recruit the specific procaspase-8 and active caspase-8 by proteolytic cleavage. Furthermore, caspase-8 is able to activate caspase-3 and caspase-7, which results in the occurrence of a caspase cascade in apoptotic cells (3). Previous studies have indicated that the apoptotic cells or immunogenic cell death (ICD) release “find-me” signal/nucleotides, such as ATP and UTP, through the plasma membrane channel pannexin 1 (PANX1) (4–6). PANX1 has been identified as a target of effector caspases 3 and 7.

The discovery of new metabolites in apoptotic cells suggests that those cells release a variety of nucleotides or other mediators of apoptosis. Interestingly, a recent study published in *Nature* (7) showed the discovery of new “find me” signals that are released from apoptotic cells through their PANX1 channels. These signals might alter certain gene expressions in adjacent normal cells (Figure 1). The authors also showed that mixtures of metabolites from apoptotic cells could alleviate specific types of inflammation *in vivo*.

**Figure 1:**
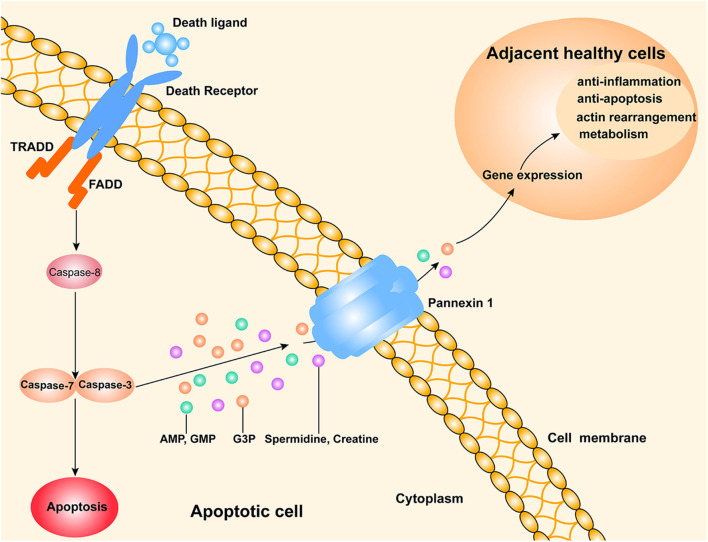
A schematic drawing of a possible mechanism by which apoptotic cells release metabolites to affect their neighboring live cells. The extrinsic pathway of apoptosis is triggered by death ligands (e.g., FasL and TNF-a) binding to death receptors. Subsequently, the adaptor FAS associated death domain protein (FADD) is recruited to activate caspase-8, which promotes a downstream caspase cascade that typically involves caspase-3 and caspase-7. PANX1, a channel for cell metabolite transport, is an effector of caspase-3 and caspase-7. Various metabolites (AMP, GMP, creatine, spermidine, and G3P) released from apoptotic cells can enter the neighboring live cells, where they regulate the expression of genes involved in several important physiological and pathological processes.

Medina et al. (7) analyzed metabolites found in the supernatants of cultured Jurkat T cells, primary mouse thymocytes, primary mouse bone-marrow-derived macrophages (BMDMs), and macrophages that had been induced by various methods to undergo caspase-dependent apoptosis *in vitro*. They showed that adenosine monophosphate (AMP), guanosine 5-monophosphate (GMP), creatine, spermidine, and glycerol-3-phosphate (G3P) were five conserved metabolites that were released from all of the apoptotic cells. Subsequently, the authors also showed that various apoptotic tumor cell lines such as Jurkat cell A549 lung epithelial cells and HCT116 colonic epithelial cells contained high levels of ATP, spermidine, G3P, and creatine. Moreover, they found that specific channels PANX1 was indispensable for releasing soluble factors through the membranes of apoptotic cells.

The authors examined whether metabolites released from apoptotic cells might influence gene expressions. RNA sequencing was performed to evaluate the expressions of genes in adjacent normal cells. They found that metabolites released by apoptotic cells could act as signaling molecules that altered gene expressions in adjacent normal cells. Furthermore, they also found that the genes with altered transcription were involved in actin rearrangements, inflammation, wound healing, and tissue repair, which are anti-apoptotic functions. To further study the significance of the metabolites, the authors established two inflammation models, including an inflammatory arthritis and a lung-transplant rejection. Interestingly, administration of the metabolite mixtures not only helped to reduce several parameters of arthritis, but also reduced inflammation in the mouse model of lung transplantation. Likewise, during sepsis-induced apoptosis of immune cells (8), the released anti-inflammatory cytokines could impair the inflammation-induced early carcinogenesis of colorectal cancer through the expansion of intestinal Treg cells (9). Therefore, these findings indicated that apoptosis cells were able to produce anti-inflammatory mediators and inhibit inflammation. However, a very recent study demonstrated that cytotoxic CD8^+^ T cells mediated ICD through the caspase-3 dependent pathway on cancer cells to generate pro-inflammatory factors which is involved in elimination of tumor cells (8). Thus, these apparent contradictory findings suggest that anti-inflammatory properties of apoptosis might be context dependent, and designed strategies to use apoptosis as anti-inflammatory therapy would require a major understanding of the mechanisms involved and likely specific analyses in every disease.

Exciting new findings always generate a wealth of avenues for future investigation, and the work of Medina et al. is no exception. First, previous study suggest that the ICD form of apoptosis released damage-associated molecular patterns (DAMPs), such as calreticulin(CRT), ATP, heat shock proteins 70 (HSP70), interleukin (IL)-1β, type I interferon (IFN), and high mobility group box 1(HMGB1) during anticancer immunity (10, 11). Therefore, other forms of programmed cell death may cause the release of specific metabolites that act as “find me” signals, either through PANX1 channels or numerous other channels. Second, it still must be determined how metabolites from apoptotic cells enter into adjacent normal cells, and also how those metabolites exert their diverse physiological affects; some possibilities include receptor-ligand binding and a specific signaling pathway. Third, it is possible that metabolite mixtures found in certain inflammatory environments or spatiotemporal relationships can induce degenerative diseases. It remains to be further explored whether these metabolite mixtures can be used to reduce the severity of autoimmune disorders or help treat tumors. If so, the discovery made by Medina et al. and its implications in treating inflammation or tumors have far-reaching consequences.

Taken together, this is a ground-breaking study of apoptotic cells, as it not only lends further support to the notion that apoptotic cells may release nucleotides, but also reveals the presence of some novel components in apoptotic cells, i.e., their metabolites. These new findings provide a novel insight into the fundamental biochemistry of apoptotic cells, and significantly contribute to developing new therapies for diseases.

## Author Contributions

CZ designed and drafted the manuscript. JL, ZS, and HP drew Mechanism chart. FX reviewed and revised the manuscript. All authors read and approved the final manuscript.

## Conflict of Interest

The authors declare that the research was conducted in the absence of any commercial or financial relationships that could be construed as a potential conflict of interest.

## Abbreviations

TNF-α: tumor necrosis factor-α
FADD: Fas-associated protein with death domain
TRADD: tumor necrosis factor receptor 1-associated death domain protein
FASLs: Fas ligands
PANX1: pannexin 1
AMP: adenosine monophosphate
GMP: guanosine 5-monophosphate
G3P: glycerol-3-phosphate
ICD: immunogenic cell death
DAMPs: damage-associated molecular patterns
CRT: calreticulin
HSP70: heat shock proteins 70
HMGB1: high mobility group box 1.
